# General Principles for Yield Optimization of Nucleoside Phosphorylase‐Catalyzed Transglycosylations†

**DOI:** 10.1002/cbic.201900740

**Published:** 2020-01-28

**Authors:** Felix Kaspar, Robert T. Giessmann, Katja F. Hellendahl, Peter Neubauer, Anke Wagner, Matthias Gimpel

**Affiliations:** ^1^ BioNukleo GmbH Ackerstrasse 76 13355 Berlin Germany; ^2^ Department of Biotechnology Technical University of Berlin ACK24, Ackerstrasse 76 13355 Berlin Germany

**Keywords:** equilibrium constant, nucleosides, nucleoside phosphorylases, pentose-1-phosphate, phosphates

## Abstract

The biocatalytic synthesis of natural and modified nucleosides with nucleoside phosphorylases offers the protecting‐group‐free direct glycosylation of free nucleobases in transglycosylation reactions. This contribution presents guiding principles for nucleoside phosphorylase‐mediated transglycosylations alongside mathematical tools for straightforward yield optimization. We illustrate how product yields in these reactions can easily be estimated and optimized using the equilibrium constants of phosphorolysis of the nucleosides involved. Furthermore, the varying negative effects of phosphate on transglycosylation yields are demonstrated theoretically and experimentally with several examples. Practical considerations for these reactions from a synthetic perspective are presented, as well as freely available tools that serve to facilitate a reliable choice of reaction conditions to achieve maximum product yields in nucleoside transglycosylation reactions.

## Graphical abstract

**Not all nucleosides are created equal**: The equilibrium constants of phosphorolysis of different nucleosides vary drastically, and so does their yield and behavior in biocatalytic transglycosylation reactions. The data, mathematical tools and guidelines presented here facilitate the reliable choice of reaction conditions for maximum yield in these reactions.

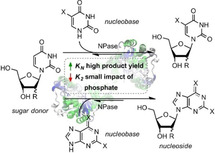

Nucleosides are highly functionalized biomolecules essential for the storage of information as DNA and RNA, cellular energy transfer and as enzyme cofactors. Modified nucleosides are widely employed as pharmaceuticals for the treatment of cancers and viral infections.^1^ Consequently, their synthetic accessibility is crucial. However, the preparation of nucleosides and nucleoside analogues by conventional synthetic methods heavily relies on protecting groups and, thus, suffers from poor atomic efficiency and low yields.^2^, ^3^, ^4^, ^5^

Biocatalytic methods offer the efficient and protecting group‐free synthesis of pyrimidine and purine nucleosides. The use of nucleoside phosphorylases (NPases) for the preparation of nucleosides and their analogues in transglycosylation reactions is firmly established^6^ and numerous examples of enzymatic or chemoenzymatic syntheses can be found in the literature.^7^, ^8^, ^9^, ^10^, ^11^, ^12^, ^13^, ^14^ NPases catalyze the reversible phosphorolysis of nucleosides to pentose‐1‐phosphates (Scheme 1, I). In transglycosylation reactions, a forward and a reverse nucleoside phosphorolysis are coupled in situ to glycosylate a free nucleobase with the pentose‐1‐phosphate generated by the first reaction (Scheme 1, I and II). Formally, this equals a direct glycosylation of the nucleobase to yield a nucleoside of interest. Conveniently, nature has provided an arsenal of robust biocatalysts that offer a broad substrate spectrum, excellent tolerance to harsh reaction conditions as well as perfect regio‐ and diastereoselectivity at the C1′ position.^11^, ^12^

**Scheme 1:**
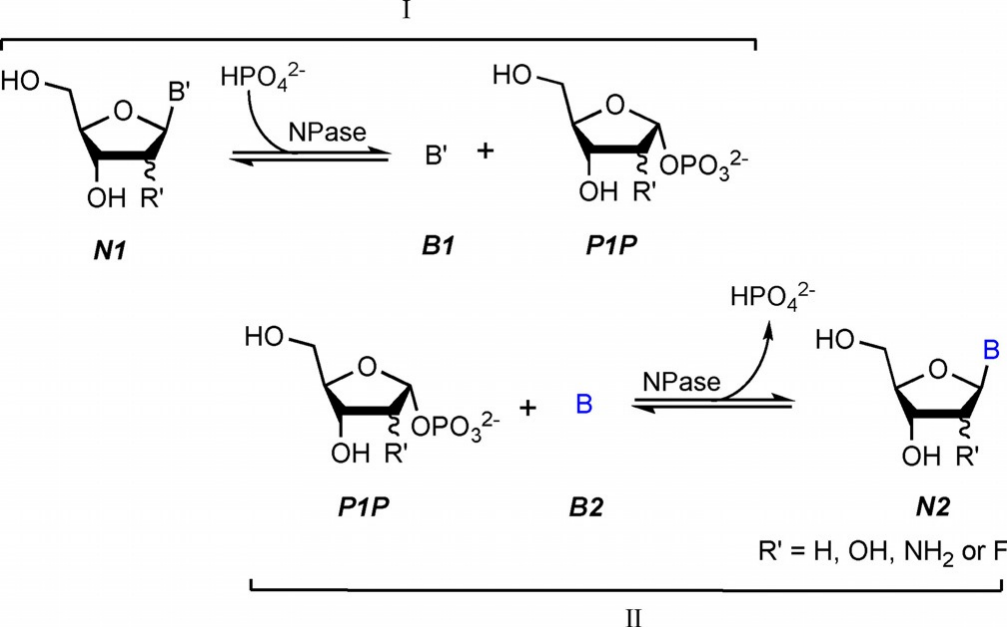
Reaction sequence of a nucleoside transglycosylation.

Despite their great versatility, enzymatically catalyzed nucleoside transglycosylation reactions have previously suffered from an unclear interrelation between yields and the employed enzymes and starting materials. Particularly, the impact of different sugar donors and/or nucleobases as well as varying phosphate concentrations on the product yield had remained unclear until recently. The pioneering work of Alexeev et al.^15^ demonstrated that yields of nucleoside transglycosylation reactions involving uridine and adenosine can be accurately predicted based on the equilibrium constants of phosphorolysis of the sugar donor and the product nucleoside. They concluded that the ratio of the equilibrium constants of the sugar donor and the product nucleoside (*K*_1_/*K*_2_) determines maximum product yields and that an excess of sugar donor is further beneficial. On the other hand, increasing phosphate concentrations were shown to have a negative impact on product yields. However, Alexeev and colleagues^15^ based their calculations on the assumption that the concentration of phosphate is constant and furthermore only investigated one example of a high‐yielding NPase‐catalyzed transglycosylation. As a continuation of the considerations of Alexeev et al.^15^ we explored this reaction system from a practical synthetic perspective and developed a universally applicable equation for yield prediction. We show that the yield‐diminishing effect of phosphate strongly depends on the equilibrium constant of phosphorolysis of the nucleoside of interest (*K*_2_). This important feature, which proved critical in the biocatalytic preparation of pharmaceutically relevant pyrimidine nucleosides has thus far not been described theoretically or experimentally. Alongside our freely available Python code for precise yield predictions (see below) we also provide a simplified equation for the estimation of product yield that allows for straightforward analytical solutions instead of the numerical solutions previously required.

Nucleoside transglycosylation reactions are generally considered as formal glycosylations of a nucleobase **B2**, which yields the corresponding nucleoside of interest, **N2**. Here, a starting nucleoside, **N1**, is used as a glycosylation agent with the purpose of donating the sugar moiety. In an enzyme cascade, the sugar donor **N1** is subjected to phosphorolysis yielding a pentose‐1‐phosphate (**P1P**), which is consumed in the sequential reaction with nucleobase **B2** to produce **N2** (Scheme 1). The yield of this reaction is generally defined as the formation of **N2** in respect to **B2**, neglecting the other reagents **P1P**, **B1**, **N1** and phosphate. Indeed, inorganic phosphate only plays a catalytic role as it is used in the first step but liberated again in the following reaction.

Generally, yields in NPase‐catalyzed transglycosylations are dictated by the equilibrium constraints of the two half reactions I and II [Eqs. (1), (2)]:

(1) $$K{}_{1}=\frac{\left[B1][P1P\right]}{\left[N1][P\right]}$$

(2) $$K{}_{2}=\frac{\left[B2][P1P\right]}{\left[N2][P\right]}$$

where *K*_1_ and *K*_2_ are the apparent equilibrium constants of phosphorolysis of the sugar donor and product nucleoside, respectively, [P] is the equilibrium concentration of phosphate, [P1P] is the equilibrium concentration of the pentose‐1‐phosphate and [N1], [N2], [B1], and [B2] are the equilibrium concentrations of the nucleosides and bases. Alexeev et al.^15^ previously solved this system of equations by assuming a constant concentration of phosphate and numerically solving the resulting cubic equation.

When we attempted to apply their equations to the synthesis of the pharmaceutically relevant nucleoside 5‐ethynyluridine we were unable to obtain results that were in agreement with experimental HPLC data, as their formula yielded negative values for this case (Table S1 in the Supporting Information). Therefore, we sought to establish a mathematical tool that allows general applicability and reaction optimization of all nucleoside transglycosylations. Bypassing the simplification made by Alexeev and co‐workers, we implemented the system of equilibrium constraints (1) and (2) including all reagents as variables in a Python code to obtain more precise predictions (see externally hosted Python code).^16^

Numerical solutions of this system allowed theoretical examination of the effect of phosphate and sugar donor excess on the product yield, considering a reasonable range of equilibrium constants.^17^ Approaching zero phosphate concentration, the maximum (ideal) product yield can be obtained, but at higher phosphate concentrations an apparent loss of yield can be observed due to phosphorolysis (or non‐synthesis) of the product nucleoside (Figure 1). Whereas the *K*_1_/*K*_2_ ratio (equal to *K*_N_) dictates the maximum yield with minimal phosphate, *K*_2_ determines the extent of yield loss in the presence of phosphate. A high *K*_N_ in the order of 5–15 promises good to excellent yields (i.e., >90 %) with only moderate excesses (i.e., twofold) of the sugar donor. On the other hand, reactions with a low *K*_N_ require a great excess of sugar donor to facilitate yields upward of 50 %. Interestingly, the effect of phosphate varies between systems with the same *K*_N_, which results from the fact that high *K*_2_ values dictate a greater degree of phosphorolysis of **N2** at non‐negligible phosphate concentrations—even at great excess of the sugar donor **N1** (Figure 1). Notably, whereas potential formation of intermediate pentose‐1‐phosphate needs to be considered for a realistic assessment and prediction of synthetic yield, we only observed less than four percentage points of deviation from the ideal yield for any nucleoside transglycosylation with <0.3 equiv of phosphate in the reaction conditions we covered with our considerations.^16^

**Figure 1:**
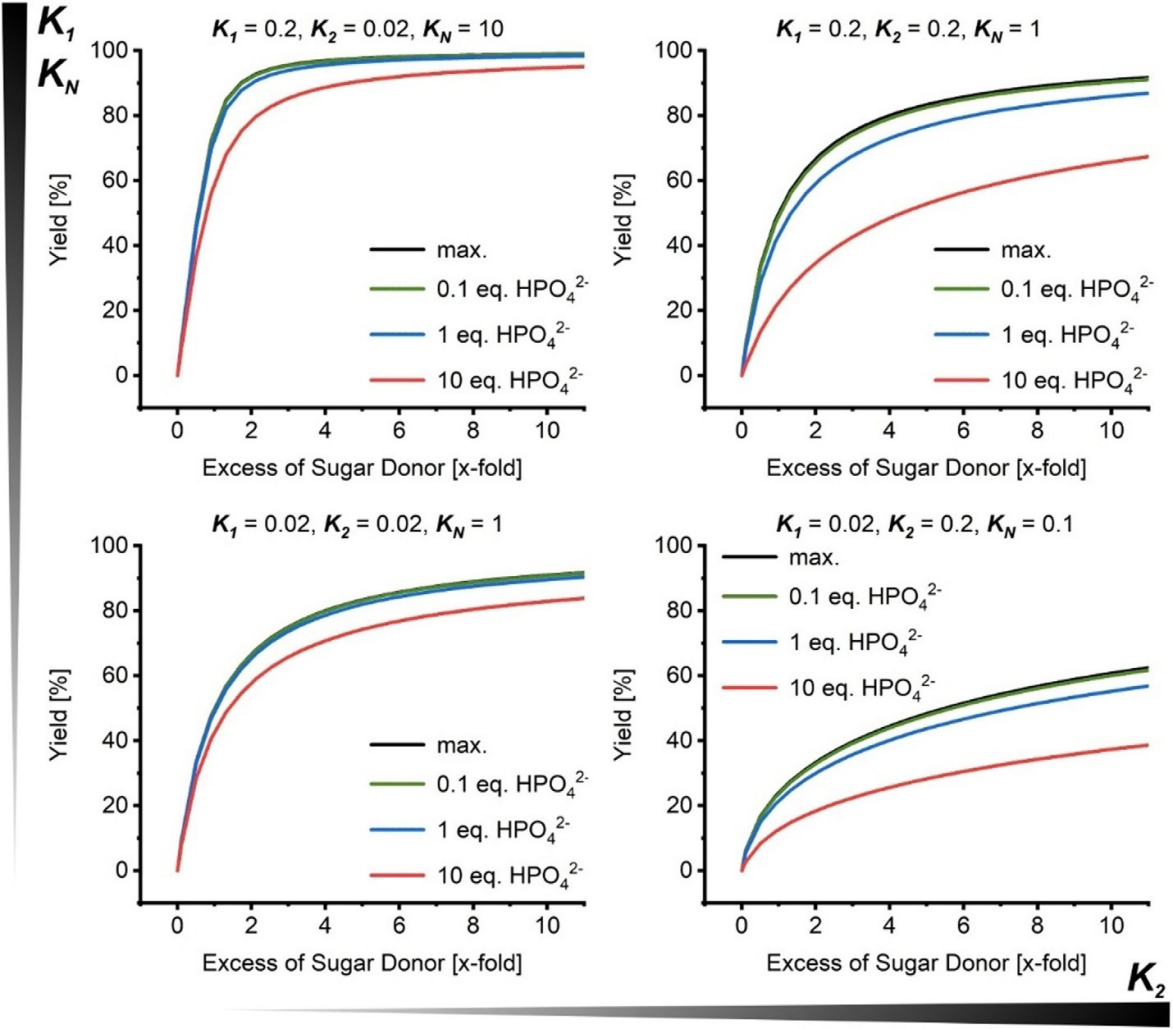
Impact of different *K*_1_ and *K*_2_ values on transglycosylation yield and phosphate gap. Realistic *K*_1_ and *K*_2_ values were assumed based on recently reported equilibrium constants.^17^ The graphs for maximum yield (max.; black), 0.1 equiv (green), 1 equiv (blue) and 10 equiv (red) of phosphate were plotted using numerical solutions of the system of equilibrium constraints (1) and (2) calculated with the Python code described in the external Supporting Information.^16^

To validate these predictions experimentally and demonstrate the varying impact of phosphate on the product yield, we prepared a series of natural and base‐modified ribosyl nucleosides from their respective nucleobases, using uridine as a sugar donor. Fitting of the experimental data to the equilibrium constraints^15^, ^16^ yielded equilibrium constants *K*_1_ and *K*_2_ very similar to those reported previously^17^ and revealed a great range of apparent equilibrium constants *K*_2_ (0.01 to 0.35 at 60 °C, pH 9) and *K*_N_ (0.4 to 16.0). In all cases, the experimental yields determined by HPLC agreed well with the predictions obtained for different phosphate concentrations (Figure 2). Our data emphasize that, as illustrated in Figure 1, particularly the yields of transglycosylation reactions with high *K*_2_ values suffer enormously from phosphate concentrations higher than strictly necessary. For instance, adenosine formation (*K*_2_=0.01) was impacted only minorly by the addition of 10 equiv of phosphate (92 % ideal yield vs. 88 % experimental yield with 10 equiv of phosphate), but 5‐ethynyluridine yield (*K*_2_=0.35) dropped by more than 30 percentage points under the same conditions (53 % ideal yield vs. 22 % experimental yield with 10 equiv of phosphate; Figure 2). Thus, steep losses in yield should be expected for products with a high *K* value (*K*_2_), whereas the synthesis of nucleosides with low *K* values tolerates significant amounts of phosphate (Figure 2). Consistent with our predictions, we only observed small deviations from the maximum yield in the experiments with 0.2 equivalents of phosphate. Thus, the concentration of phosphate should be kept low in synthetic nucleoside transglycosylations to obtain maximum yield. For these cases, calculation of maximum (ideal) conversion provides a close approximation of the yield and allows for the use of a simplified formula.

**Figure 2:**
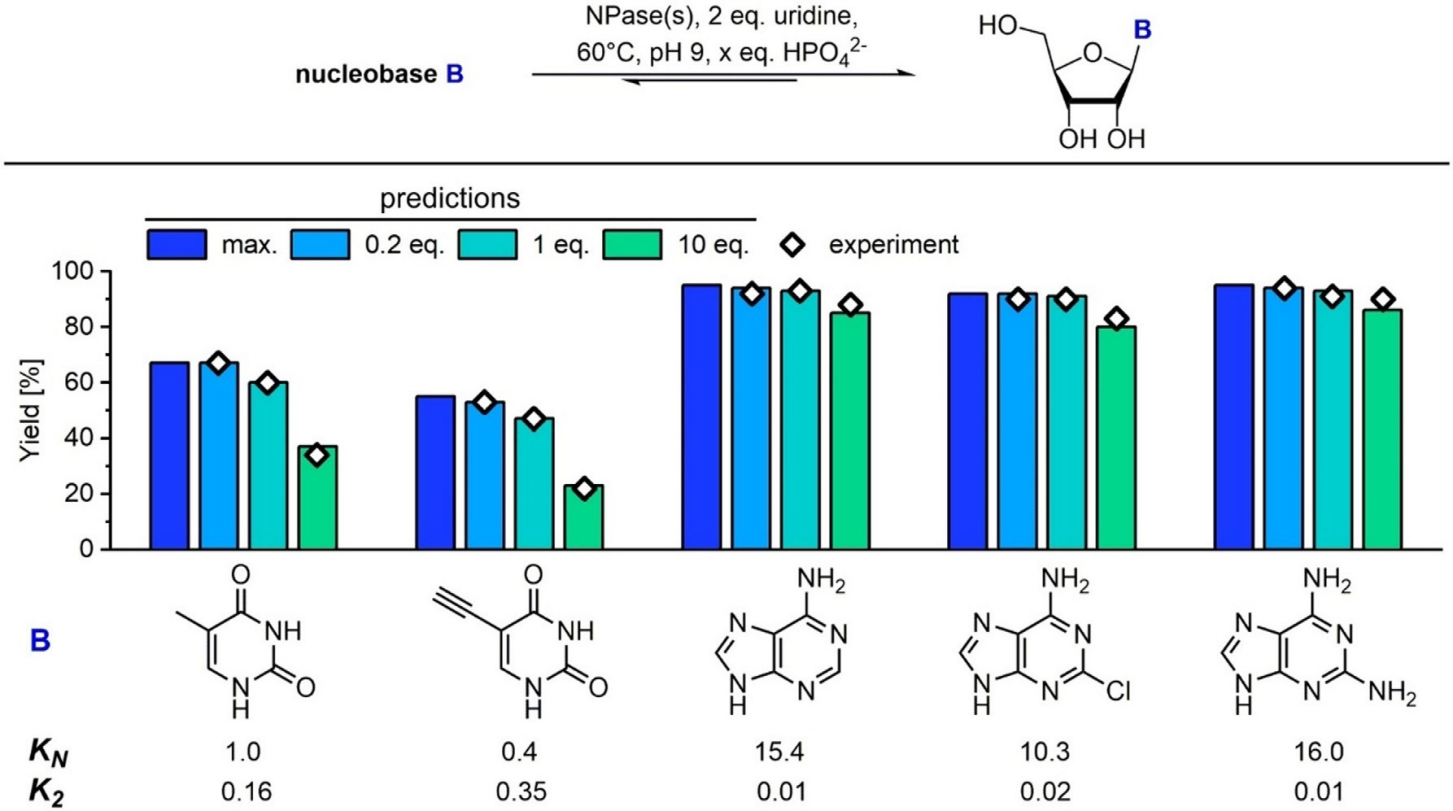
Biocatalytic synthesis of nucleosides by transglycosylation. Reactions were performed with 1 mm uridine as sugar donor (*K*_1_=0.16), 0.5 mm nucleobase, 32 μg mL^−1^ pyrimidine NPase (2.5 U mL^−1^) and 66 μg mL^−1^ purine NPase (5.0 U mL^−1^) in 50 mm glycine buffer at pH 9 and 60 °C with either 0.1 mm (0.2 equiv in respect to the starting base), 0.5 mm (1 equiv) or 5 mm (10 equiv) K_2_HPO_4_ in a total volume of 1 mL. Experimental yield (◊) was determined by HPLC considering conversion of the free nucleobase to its corresponding ribosyl nucleoside. Predictions (blue, light blue, turquoise and green columns) were carried out with the Python code described in the external Supporting Information.^[16]^ The values for the maximum yield (max.; blue) can also be obtained from Equation (4).

Considering ideal (intermediate‐free) coupling of the two half reactions, I and II, the terms for phosphate and **P1P** would cancel in the mathematical consideration of this system, as the production of these in one half reaction is compensated by the consumption in the other. Considering the net reaction, one may therefore define [Eq. (3)]:

(3) $$K{}_{N}=\frac{K{}_{1}}{K{}_{2}}=\frac{\left[N2][B1\right]}{\left[N1][B2\right]}$$

with definitions from above. Solving this equation for the concentration of the product nucleoside, **N2**, yields only one physically possible solution that can be used to calculate ideal (phosphate‐ and **P1P**‐free) yields of nucleoside transglycosylations with variable initial concentrations of the sugar donor **N1** and the nucleobase **B2**, [**N1**]_0_ and [**B2**]_0_, respectively [Eq. (4)]:

(4) [N2]=KN([N1]0+[B2]0)2(KN-1)-√KN(KN[N1]20-2KN[N1]0[B2]0+KN[B2]20+4[N1]0[B2]0)2(KN-1)

Thus, the maximum yield (at zero phosphate) can be calculated easily from Equation (4) to reflect a realistic estimate of the experimental yield if <0.3 equiv of phosphate are used. Ideal yields for conversions employing a range of pyrimidine and purine ribosyl and 2′‐deoxyribosyl nucleosides^17^ with different reaction conditions including sugar donor excess and temperature, can be calculated with an Excel sheet freely available from the externally hosted Supporting Information.^18^

These considerations and previous findings^15^, ^16^ bear several practical implications for NPase‐catalyzed nucleoside transglycosylations. First, a high *K*_1_/*K*_2_ ratio (high *K*_N_) leads to excellent yields which can be achieved with moderate excess of the sugar donor, as mentioned by Alexeev and colleagues,^15^ and estimated easily with Equation (4). Second, pyrimidine nucleosides serve better as sugar donors than purine nucleosides.^17^ From a practical point of view, uridine and thymidine recommend themselves as ribosyl and 2′‐deoxyribosyl donor, respectively, due to their simple commercial availability and high *K* value. Third, phosphate concentration in the transglycosylation reaction should generally be kept as low as possible to prevent loss of product yield. This becomes especially important in the synthesis of nucleosides with high *K* values, such as pyrimidine nucleosides like 5‐ethynyluridine. Thus, 0.1–0.3 equivalents of phosphate in respect to the starting base may present an appropriate trade‐off between reaction speed and maximum yield. A potential workflow for the fruitful application of the methodology presented in this work is suggested in the Supporting Information.

Given the easy accessibility of apparent equilibrium constants of phosphorolysis of any nucleoside of interest, the tools for yield prediction presented in this work aid the straightforward design and optimization of nucleoside transglycosylations to facilitate high yields in NPase‐catalyzed reactions. Exact yield prediction of transglycosylations may be performed with our Phyton code considering phosphate^16^ and practical estimations for ideal yield can easily be obtained from Equation (4).^18^

## Experimental Section

Enzymatic nucleoside transglycosylations were performed with 0.5 mm nucleobase, 1 mm uridine as sugar donor, 32 μg mL^−1^ pyrimidine NPase (2.5 U mL^−1^; E‐PyNP‐0002, BioNukleo GmbH, Berlin, Germany) and 66 μg mL^−1^ purine NPase (5.0 U mL^−1^; E‐PNP‐0002, BioNukleo GmbH) in 50 mm glycine buffer at pH 9 and 60 °C with either 0.1 mm (0.2 equivalents in respect to the starting base), 0.5 mm (1 equiv) or 5 mm (10 equiv) K_2_HPO_4_ in a total volume of 1 mL. Reaction mixtures were prepared from stock solutions and started by the addition of the enzyme(s). Time to equilibrium was approximated via UV/Vis spectroscopy.^19^ Allowing for additional time after apparent reaction completion, the reactions were stopped after 1 h by quenching samples of 100 μL in an equal volume of MeOH and analyzed by HPLC. All experimental and calculated data are available online.^16^, ^18^

## Conflict of interest

*The authors declare no conflict of interest*.

## Supporting information

As a service to our authors and readers, this journal provides supporting information supplied by the authors. Such materials are peer reviewed and may be re‐organized for online delivery, but are not copy‐edited or typeset. Technical support issues arising from supporting information (other than missing files) should be addressed to the authors.

Supplementary
