# Supplementary material for: General Principles for Yield Optimization of Nucleoside Phosphorylase‐Catalyzed Transglycosylations
Source: Chembiochem. 2020 Jan 28;21(10):1428–32. doi: 10.1002/cbic.201900740 (PMC7318676; doi:10.1002/cbic.201900740)
Supplement: Supplementary file 1 — Supplementary [file CBIC-21-1428-s001.pdf]

CHEMBIOCHEM

Supporting Information

General Principles for Yield Optimization of Nucleoside Phosphorylase-Catalyzed Transglycosylations**

Felix Kaspar+,*[a] Robert T. Giessmann+,[b] Katja F. Hellendahl,[b] Peter Neubauer,[b] Anke Wagner,*[a, b] and Matthias Gimpel[b]

cbic_201900740_sm_miscellaneous_information.pdf

Author Contributions

F.K. Conceptualization: Equal; Data curation: Equal; Formal analysis: Lead; Investigation: Lead; Methodology: Equal; Project administration: Equal; Visualization: Lead; Writing - Original Draft: Lead; Writing - Review & Editing: Lead
R.G. Conceptualization: Equal; Data curation: Equal; Formal analysis: Equal; Funding acquisition: Equal; Methodology: Equal; Project administration: Equal; Resources: Equal; Software: Lead; Supervision: Lead; Writing - Original Draft: Supporting; Writing - Review & Editing: Supporting
K.H. Investigation: Equal; Writing - Review & Editing: Supporting
P.N. Conceptualization: Supporting; Funding acquisition: Lead; Resources: Lead; Supervision: Equal; Writing - Review & Editing: Supporting
A.W. Conceptualization: Supporting; Funding acquisition: Equal; Project administration: Equal; Resources: Equal; Supervision: Equal; Writing - Review & Editing: Equal
M.G. Conceptualization: Supporting; Supervision: Equal; Writing - Review & Editing: Supporting.

Supplementary Information

Author contributions 1
Conflict of Interest 1
Sample analysis by HPLC 2
Comparison of HPLC data and Calculations with Alexeev's formula 2
Graphical Representation of the Solutions with Alexeev's formula 3
Table S2. HPLC data. 4
Suggested workflow for NPase-catalyzed nucleoside synthesis 5
References 6

Author contributions (with definitions as recommended by Brand *et al.*[1])

Conceptualization, F.K., R.T.G., P.N., A.W. and M.G.; Data curation, F.K. and R.T.G.; Formal analysis, F.K. and R.T.G.; Funding acquisition, R.T.G., P.N. and A.W.; Investigation, F.K. and K.H.; Methodology, F.K. and R.T.G.; Project administration, F.K., R.T.G. and A.W.; Resources, R.T.G., K.H., A.W. and P.N.; Software, R.T.G. ; Supervision, R.T.G., P.N. A.W. and M.G.; Validation, - ; Visualization, F.K.; Writing—original draft, F.K. and R.T.G.; Writing—review & editing, F.K., R.T.G., K.H., P.N., A.W. and M.G.

Conflict of Interest

A.W. is CEO of the biotech company BioNukleo GmbH. F.K. is a researcher at BioNukleo GmbH and P.N. is a member of the advisory board.

Sample analysis by HPLC

HPLC analysis was carried out with an Agilent 1200 Series HPLC System, employing a Phenomenex Kinetex Evo C18 100 Å column (250 mm x 4.6 mm). Separation was achieved using an isocratic elution at 3% MeCN in 20 mM NH_4Ac for 5 min followed by a linear gradient of 3—40% MeCN in 20 mM NH_4Ac over 20 min. The typical retention times for the nucleosides and bases in this work were 4.2 min (2-aminoadenine), 4.4 min (5-methyluracil), 4.6 min (5-ethynyluracil), 5.1 min (adenine), 5.6 min (5-methyluridine), 7.5 min (5-ethynyluridine), 9.3 min (2-aminoadenosine), 10.3 min (adenosine), 10.9 min (2-chloroadenine) and 13.2 min (2-chloroadenosine). For the calculation of concentration from peak area, calibration coefficients α [$\mu M \cdot mV^{-1} \cdot s^{-1}$] of 0.5625 (5-methyluracil), 0.3693 (5-methyluridine), 0.7267 (5-ethynyluracil), 0.9091 (5-ethynyluridine), 0.2680 (adenine), 0.2486 (adenosine), 0.2738 (2-chloroadenine), 0.2515 (2-chloroadenosine), 0.6150 (2-aminoadenine) and 0.3758 (2-aminoadenosine) were used.

Yield (Y) was calculated as

$$Y = \frac{P}{S + P}$$

where S and P are the concentrations determined from HPLC peak areas of the substrate and the product, respectively.

Table S1. Comparison of HPLC data and Calculations with Alexeev's formula[3] for the synthesis of 5-ethynyluridine by transglycosylation[a]

Phosphate [eq.]	HPLC yield [%]	Calculated yield [%] Alexeev's formula
0.2	53	34
1	47	-6
10	22	-271

[a] We used reaction conditions of 0.5 mM 5-ethynyluracil, 1 mM uridine as sugar donor, x equivalents of phosphate in respect to the starting base, pH 9 and 60 °C. Reactions were performed as described in the Experimental section. Previously reported[4] equilibrium constants of K_1 = 0.22 and K_2 = 0.89 were used for calculation.

Graphical Representation of the Solutions with Alexeev's[3] formula

Numerical solutions of the cubic equation provided by Alexeev can be obtained as the best fit of an approximated x-value to solve the equation, namely provide a value of zero. Thus, the best solutions is represented by the x-value (in this case equal to the conversion of the sugar donor, N1) for which the function intersects the horizontal axis (vale of the cubic equation = 0). As evident from Figure S1, only one of these values exists for each set of input variables. However, the yield obtained via these solutions (as a function of a further equation provided by Alexeev) is not realistic.

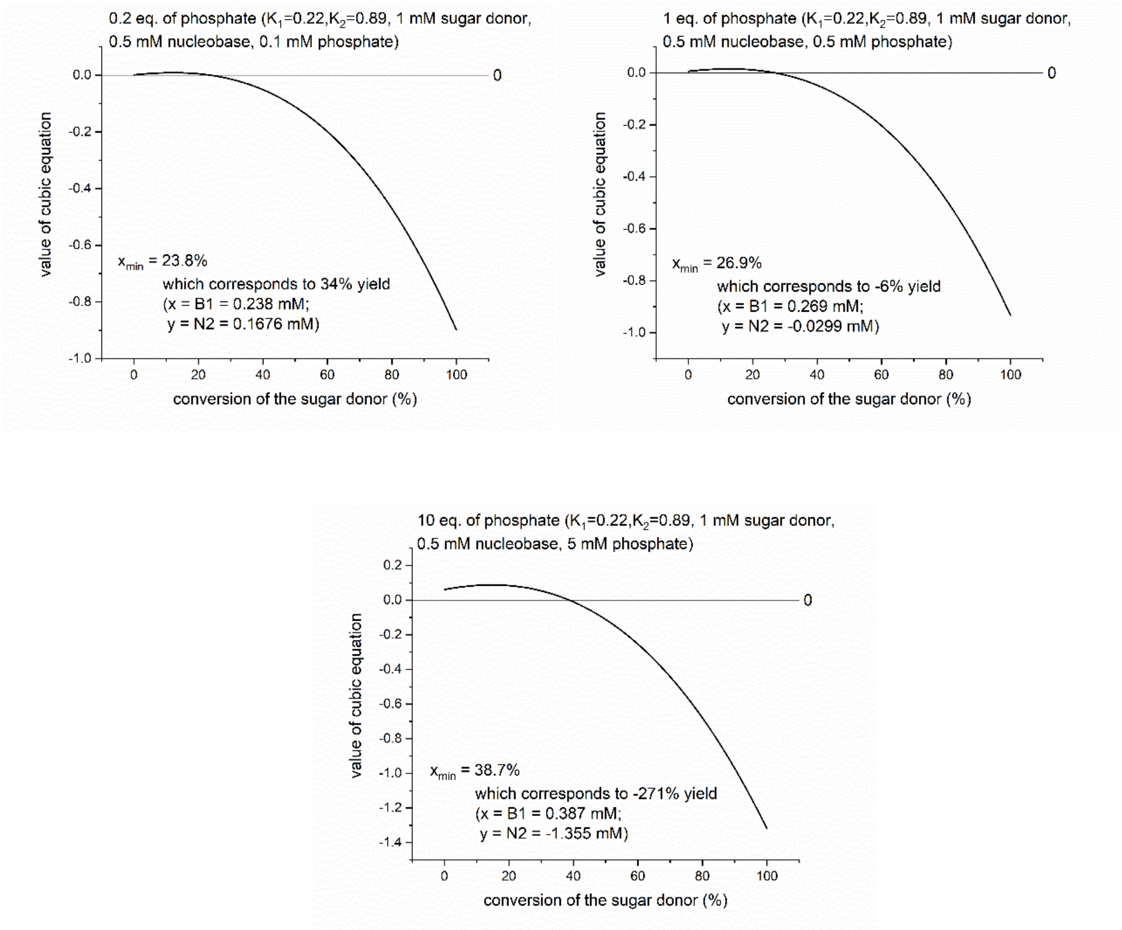

Figure S1. Numerical solutions of Alexeev's[3] formula (equation (1) in their manuscript). Yield was calculated with equation (2) from the report of Alexeev.

Table S2. HPLC data

product	phosphate [equivalents]	peak area [mVs]		concentration [mM]		Yield of ***N2*** [%]
		N2	***B2***	***N2***	***B2***	
5-methyl-uridine	0.2	689	224	0.254	0.126	66.9
	1	686	294	0.253	0.165	60.5
	10	411	524	0.152	0.295	34.0
5-ethynyl-uridine	0.2	277	308	0.252	0.224	52.9
	1	265	369	0.241	0.268	47.3
	10	115	522	0.105	0.379	21.6
adenosine	0.2	1811	152	0.450	0.041	91.7
	1	1913	137	0.476	0.037	92.8
	10	1769	231	0.440	0.062	87.7
2-chloro-adenosine	0.2	1826	191	0.459	0.052	89.8
	1	1887	189	0.475	0.052	90.2
	10	1719	330	0.432	0.090	82.7
2-amino-adenosine	0.2	1103	41	0.414	0.025	94.3
	1	1125	67	0.423	0.041	91.1
	10	1083	76	0.407	0.047	89.7

Suggested workflow for NPase-catalyzed nucleoside synthesis

For maximal yields in NPase-mediated transglycosylations, phosphate should always be kept minimal. Therefore, we recommend abstaining from adding more than catalytic amounts of phosphate to reaction mixtures (e.g. 0.1 equivalents in respect to the starting base).

Step 1: Selection of the sugar donor

A sugar donor should be selected based on price and equilibrium constant of phosphorolysis. Ideally, K_1 should be as high as possible. If, however, price prevents the use of the "ideal" sugar donor, a trade-off needs to be made to ensure profitability, since, in most cases, the sugar donor needs to be applied in excess to grant satisfactory yields. Additionally, solubility needs to be considered and reaction conditions may need to be adjusted. Generally, we recommend uridine and thymidine, or, if available, 7-methylated guanosyl nucleosides.

Step 2: Determination of the equilibrium constant of phosphorolysis of the product nucleoside (K_2)

If the nucleoside of interest is available in pure form, the equilibrium constant of phosphorolysis K_2 can easily be determined in a phosphorolysis experiment, monitored either by HPLC or UV spectroscopy.[2] Here, the phosphate concentration should be selected so that between 20 and 80% conversion of the nucleoside are observed to obtain reliable results. Equilibrium should be clearly visible and potential decay of reaction products (pentose-1-phosphates) should be considered if either no equilibrium is observable or apparent conversion is visible after an initial quick equilibrium. To ensure pentose-1-phosphate stability, it may be recommended to perform these experiments, as well as transglycosylation reactions, at alkaline pH. However, if the working space of the enzyme precludes application at pH 8—9, phosphorolysis may be performed under the same conditions (temperature, pH) as the anticipated transglycosylation.

If the nucleoside of interest is not at hand, K_2 may also be estimated by transglycosylation if the corresponding nucleobase is available. In a transglycosylation employing 1 eq. of sugar donor and 0.1 eq. of phosphate, the yield can be approximated and K_2 can be estimated with equation (3), if K_1 is known (if K_1 is unknown, see above).

Step 3: Consideration of the reaction conditions

Using equation (4), the required sugar donor excess to achieve satisfactory yields can be calculated. In some cases 2—3 eq. of sugar donor are sufficient to reach >90% yield, in other cases as much as 20 eq. may be needed. In the latter case, settling for less yield or the use of a different sugar donor may need to be considered.

Step 4: The Transglycosylation

The concentration of the main reactants (sugar donor, base) can be adjusted as desired as confirmed by Alexeev and colleagues,[3] as long as 1) their ratio is maintained and 2) the solubility of the reactants

allow scaling up of the reaction (e.g. the reaction can be carried out on a 2 mM or 50 mM scale, as long as all components are soluble).

References

[1] A. Brand, L. Allen, M. Altman, M. Hlava, J. Scott, *Learn. Publ.* **2015**, *28*, 151–155.

[2] F. Kaspar, R. T. Giessmann, N. Krausch, P. Neubauer, A. Wagner, M. Gimpel, *Methods Protoc.* **2019**, *2*, 60.

[3] C. S. Alexeev, I. V Kulikova, S. Gavryushov, V. I. Tararov, S. N. Mikhailov, *Adv. Synth. Catal.* **2018**, *360*, 3090–3096.

[4] F. Kaspar, R. T. Giessmann, P. Neubauer, A. Wagner, M. Gimpel, *Adv. Synth. Catal*. **2019**, accepted article.
